# Evaluation of Antimicrobial Susceptibility and Resistance Patterns of Treponema denticola Isolated From Periodontal Disease: An In Vitro Study

**DOI:** 10.7759/cureus.61497

**Published:** 2024-06-01

**Authors:** Amit R Pawar, Jaiganesh Ramamurthy, A. S. Smiline Girija

**Affiliations:** 1 Department of Periodontics, Saveetha Dental College and Hospitals, Saveetha Institute of Medical and Technical Sciences, Saveetha University, Chennai, IND; 2 Department of Microbiology, Saveetha Dental College and Hospitals, Saveetha Institute of Medical and Technical Sciences, Saveetha University, Chennai, IND

**Keywords:** red complex bacteria, treponema, resistance, plaque, periodontal disease, antimicrobial

## Abstract

Background

Periodontal disease poses a significant oral health challenge, involving inflammatory conditions impacting tooth-supporting structures. Treponema denticola, a "red complex" organism, plays a crucial role in periodontal pathogenesis, forming biofilms in subgingival environments and contributing to dysbiosis. Antimicrobial therapy is pivotal in managing periodontal disease, requiring a nuanced understanding of susceptibility patterns exhibited by key pathogens like T. denticola*.*

Aims and objectives

This study aims to investigate the antimicrobial susceptibility and resistance profiles of Treponema denticola, a prominent bacterium in periodontal disease, by examining its responses to various antimicrobial agents commonly used in periodontal therapy.

Methodology

Plaque samples were meticulously collected from individuals diagnosed with periodontal disease to ensure a diverse representation of the oral microbiome. All the samples were cultured, and red complex bacteria were isolated under anaerobic culture. Treponema denticola isolates were cultured from these samples under anaerobic conditions, and molecular techniques were employed for species identification. A comprehensive panel of antimicrobial agents was selected to assess the response of Treponema denticola. In vitro antimicrobial susceptibility testing (AST) was conducted using the antimicrobial gradient method, employing a hybrid approach combining elements of disk-diffusion and dilution methods.

Results

Treponema denticola had exhibited resistance to metronidazole, a commonly used antibiotic effective against anaerobic bacteria, emphasizing limitations in its applicability. However, the bacterium displayed sensitivity to tetracycline, imipenem, cefoperazone, chloramphenicol, clindamycin, and moxifloxacin, offering diverse therapeutic options. The antimicrobial gradient strip test provided detailed minimum inhibitory concentration (MIC) values, contributing to a nuanced understanding of susceptibility and resistance patterns.

Conclusion

This study significantly advances our understanding of Treponema denticola's antimicrobial susceptibility and resistance profiles in the context of periodontal disease. The findings underscore the importance of tailored treatment strategies and contribute to broader efforts in antimicrobial stewardship, aligning with global initiatives to combat antibiotic resistance. This research lays the foundation for more effective and personalized approaches to periodontal care, emphasizing the intricate microbial dynamics associated with periodontal health and disease.

## Introduction

Periodontal disease, a widespread oral health concern, encompasses a spectrum of inflammatory conditions affecting the supporting structures of the teeth [1]. Periodontal disease, or periodontitis, arises primarily due to microbial etiology, specifically the proliferation of bacterial colonies within dental plaque. The human oral cavity harbors a diverse array of microorganisms, and when oral hygiene practices are inadequate, plaque accumulates on dental surfaces. The bacterial constituents of plaque, predominantly gram-negative anaerobic species, produce pathogenic byproducts that incite an inflammatory response in the gingival tissues. Collecting subgingival plaque from periodontal pockets has been a common method to analyze changes in the microbiota associated with periodontitis [2]. Porphyromonas gingivalis, Tannerella forsythia, and Treponema denticola are extensively studied in clinical samples due to their frequent co-isolation and strong association with periodontitis [3]. These three species, known as red-complex bacteria, are recognized for their virulence and pathogenicity, contributing to chronic inflammation [4]. Managing and preventing periodontal disease requires targeting these bacteria and restoring a balanced bacterial environment in the oral cavity [5]. Among the myriad microorganisms that populate the oral cavity, Treponema denticola emerges as a significant protagonist in the intricate narrative of periodontal pathogenesis [6]. Treponemes are commonly found in the normal oral flora of humans, primarily inhabiting the subgingival region. However, they can also become established in opportunistic infections like periodontal diseases. These diseases, characterized by inflammation and tissue destruction around the tooth attachment points, are caused by anaerobic Gram-negative bacteria with proteolytic properties [7]. This spirochete bacterium has been implicated in the initiation and progression of periodontal disease due to its ability to thrive in the subgingival environment, where it forms biofilms in conjunction with other pathogenic species [8]. As a member of the oral microbiome, Treponema denticola contributes to the dysbiosis that characterises periodontal pockets, leading to chronic inflammation, tissue destruction, and, ultimately, tooth loss if left unaddressed [5-9].

The multifaceted nature of periodontal disease necessitates a comprehensive understanding of the specific microbial players involved, with Treponema denticola occupying a prominent role in this intricate ecosystem [10]. The challenge lies not only in recognizing its significance but also in devising effective strategies to combat its pathogenic potential. Antimicrobial therapy has been a cornerstone in the management of periodontal disease, aiming to disrupt and control the microbial communities responsible for the destructive inflammatory processes [11]. The effectiveness of these interventions is contingent upon a thorough understanding of the intricate patterns of antimicrobial susceptibility and resistance demonstrated by significant contributors, including Treponema denticola [12].

The isolates under examination are directly sourced from plaque samples taken from individuals experiencing periodontal disease. Through the detailed examination of how Treponema denticola reacts to different antimicrobial agents in a controlled laboratory environment, the objective is to understand the complexities of its responses to therapeutic interventions. Studies have indicated that T. denticola, along with other periodontal pathogens, can develop resistance to antibiotics commonly used in periodontal therapy. This resistance can arise through mechanisms such as genetic mutations, horizontal gene transfer, and biofilm formation, which provides a protective environment for bacteria against antimicrobial agents. This effort aims to provide essential insights for improving treatment strategies and enhancing our ability to manage the microbial dynamics associated with periodontal health and disease.

## Materials and methods

Plaque samples were meticulously collected from 30 participants diagnosed with periodontal disease (2017 Classification of Periodontal and Peri-implant Diseases and Conditions) to ensure a diverse representation of the oral microbiome. Inclusion criteria for the study required selecting participants aged 18 years and above, who provided informed consent. Only samples that showed viable growth of Treponema denticola under laboratory conditions and were confirmed through molecular techniques were included. Exclusion criteria ruled out participants who did not consent, samples that failed to grow or were contaminated, and isolates not conclusively identified as Treponema denticola. Additionally, participants who had used antibiotics or antimicrobial agents within one month prior to sample collection and individuals with systemic diseases that could impact periodontal status were excluded to ensure the accuracy and reliability of the susceptibility testing. Approval for the study was obtained from the Institutional Review Board of Saveetha Dental College and Hospital in Chennai (SRB/SDC/PERIO-2205/23/081), and all participants provided informed consent before the commencement of the research.

Using sterile instruments, samples were aseptically obtained from periodontal pockets using Gracey curette (Hu-Friedy Co, Chicago, IL, USA) and sites were selected randomly. Treponema denticola isolates were subsequently cultured from these collected plaque samples in specialized media such as fastidious anaerobe agar (FAA) and trypticase soy agar (TSA) under anaerobic conditions. Molecular techniques were employed to confirm the identification of the isolated species [13]. To assess the response of Treponema denticola to therapeutic interventions, a comprehensive panel of antimicrobial agents, reflecting those commonly used in periodontal therapy, was carefully selected. In vitro antimicrobial susceptibility testing (AST) using the antimicrobial gradient method was conducted for the isolated T. denticola strains. The gradient strip test represents a hybrid approach, combining elements of both the disk-diffusion and dilution methods in AST. AST is a crucial method in microbiology used to determine the effectiveness of antibiotics or other antimicrobial agents against specific microorganisms. First, a sample is collected from the patient, typically from the site of infection, and then cultured in a laboratory to isolate the microorganism causing the infection. Once isolated, the microorganism is identified using various biochemical, immunological, or genetic methods. The identified microorganism is then spread onto an agar plate, which is a petri dish containing a solid growth medium. Paper discs or strips impregnated with specific concentrations of antibiotics are placed onto the agar plate, and the plate is incubated at a (35±2)°C temperature for a defined period, typically 18-24 hours, allowing the bacteria to grow. After incubation, the plate is examined for zones of inhibition around each antibiotic disc. The size of the zone indicates the susceptibility of the bacteria to the antibiotic, with a larger zone indicating greater susceptibility. The diameter of the zone of inhibition is compared to standardised tables provided by organisations like the Clinical and Laboratory Standards Institute (CLSI) or the European Committee on Antimicrobial Susceptibility Testing (EUCAST) to classify the bacteria as susceptible, intermediate, or resistant to each antibiotic tested [14]. This method offers the advantageous properties of both techniques, providing a simplified yet effective means of determining the minimum inhibitory concentration (MIC) of antibiotics such as clindamycin, metronidazole, cefoperazone, moxifloxacin, chloramphenicol and tetracycline [12]. In essence, the gradient strip test operates on the principle of antibiotic diffusion through agar containing a continuous gradient. The agar medium acts as a platform for the diffusion, creating a concentration gradient of the antibiotic across its surface. This gradient allows for a range of antibiotic concentrations to be present simultaneously, making it possible to observe the point at which bacterial growth is inhibited. 

This multi-step process provides valuable insights into the susceptibility and resistance profiles of Treponema denticola, contributing to a better understanding of its interactions with antimicrobial agents commonly employed in periodontal treatment. MIC values (Table 1) provide susceptibility or resistance patterns of T. denticola. Correlations between isolates and antimicrobial agents were explored, providing insights into the variability of T. denticola responses.

**Table 1:** This table provides a clear overview of the susceptibility of T. denticola to these antimicrobial agents MIC: minimum inhibitory concentration

Antimicrobial Agent	MIC Range (µg/mL)
Clindamycin	0.25 - 1
Metronidazole	0.25 - 2
Cefoperazone	0.12 - 1
Moxifloxacin	0.125 - 2
Chloramphenicol	2 - 8
Tetracycline	0.5 - 4

The MIC values presented in Table 1 highlight the varying levels of susceptibility of Treponema denticola to different antimicrobial agents. Metronidazole, clindamycin, and moxifloxacin show generally low MIC ranges, indicating high efficacy against T. denticola. Cefoperazone also demonstrates effective inhibition with a low MIC range. Chloramphenicol has higher MIC values, suggesting moderate susceptibility, while tetracycline shows a range indicating moderate to high susceptibility.

These findings underscore the importance of selecting appropriate antimicrobial agents based on their MIC values to effectively target T. denticola. Continuous monitoring of these values is crucial to manage and mitigate potential resistance patterns in clinical settings.

## Results

In this research, we conducted a thorough examination of the susceptibility of Treponema denticola isolates to antimicrobial agents, using plaque samples obtained from individuals diagnosed with periodontal disease. Our collection of diverse plaque samples aimed to provide a representative snapshot of the oral microbiome, enabling us to specifically investigate T. denticola's response to antimicrobial agents. Through the use of specialized media and anaerobic conditions, we cultured T. denticola isolates from the collected plaque samples, and molecular techniques confirmed the accurate identification of these isolates at the species level.

**Figure 1:**
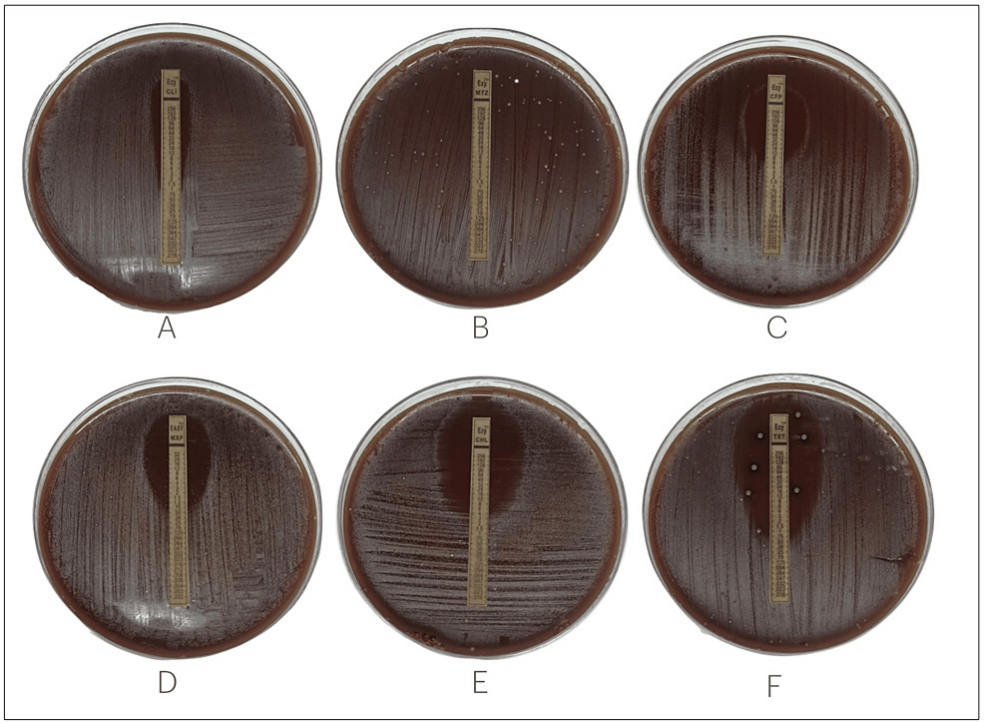
The antimicrobial gradient strip test of Treponema denticola for A) clindamycin, B) metronidazole, C) cefoperazone, D) moxifloxacin, E) chloramphenicol and F) tetracycline

The investigation revealed that T. denticola displayed resistance to metronidazole while demonstrating sensitivity to tetracycline, cefoperazone, chloramphenicol, clindamycin, and moxifloxacin (Figure 1). The resistance to metronidazole, a commonly used antibiotic effective against anaerobic bacteria, suggests that T. denticola strains in this study are not susceptible to this specific drug, highlighting the limitations of its use in this context. Conversely, the sensitivity of T. denticola to tetracycline is promising, indicating that tetracycline or related antibiotics could be effective in treating infections caused by T. denticola. Furthermore, the sensitivity to cefoperazone, chloramphenicol, clindamycin, and moxifloxacin provides a diverse set of therapeutic options. These antibiotics, belonging to different classes with distinct mechanisms of action, offer flexibility in choosing an appropriate treatment based on factors such as patient-specific considerations and the clinical context. Understanding the antimicrobial susceptibility profile of T. denticola is crucial for tailoring effective treatment strategies, optimizing therapeutic outcomes, and contributing to broader efforts in antimicrobial stewardship to combat antibiotic resistance.

## Discussion

The evaluation of antimicrobial susceptibility and resistance patterns of Treponema denticola isolated from periodontal disease is of paramount importance in understanding the dynamics of periodontal infections and optimizing treatment strategies. Treponemes like T. denticola possess mechanisms to suppress the host response to lipopolysaccharide (LPS), potentially aiding the persistence of bacterial consortia associated with periodontal disease. Additionally, T. denticola's lipooligosaccharide (LOS) can induce osteoclastogenesis and matrix metalloproteinase expression, aggravating periodontal pathology. Major virulence factors of T. denticola in chronic periodontitis include motility, chemotaxis, synergistic interactions with other pathogens, production of cytotoxic metabolites, biofilm formation, and modulation of host defense mechanisms, promoting subgingival biofilm protection and tissue destruction [15,16]. This in vitro study adds significant insights into the antimicrobial landscape of T. denticola, a bacterium intricately involved in the pathogenesis of periodontitis.

Several earlier studies have attempted to unravel the susceptibility and resistance patterns of T. denticola to various antimicrobial agents [17-21]. In a study conducted recently by Kazuko et al., 2017, doxycycline, minocycline, azithromycin, and erythromycin showed effectiveness against all tested Treponema species, while fluoroquinolones were equally effective only against T. socranskii. Kanamycin susceptibility was seen in one T. denticola strain, T. socranskii, and T. vincentii was affected by prior aerobic exposure. The susceptibility of different T. denticola strains to quinolone drugs varied [17].

Moreover, the emergence of antimicrobial resistance poses a significant challenge in the management of periodontal infections. Azithromycin's efficacy in treating periodontal bacterial biofilms lacks substantial evidence despite its growing use. The study by Ong et al. aimed to compare azithromycin with amoxicillin and metronidazole combination against biofilms containing Porphyromonas gingivalis, Treponema denticola, and Tannerella forsythia. Biofilms were cultured, treated with different concentrations of azithromycin or the combination, and assessed for biomass. Results showed the combination's superior biofilm inhibitory effect compared to azithromycin, indicating its potential clinical advantage [18].

But the investigation into the antimicrobial susceptibility and resistance profiles of Treponema denticola in this study isolated from plaque samples collected from individuals diagnosed with periodontal disease, provides valuable insights into the intricate dynamics of periodontal health and disease. The significant findings, particularly the resistance of T. denticola to metronidazole and its sensitivity to tetracycline, imipenem, cefoperazone, chloramphenicol, clindamycin, and moxifloxacin, have profound implications for periodontal treatment strategies.

The resistance observed to metronidazole is noteworthy, as this antibiotic is commonly employed in the management of anaerobic infections, including those associated with periodontal disease. The resistance of T. denticola to metronidazole emphasizes the importance of understanding the specific antimicrobial susceptibility patterns of key periodontal pathogens [19,20]. This knowledge is crucial for avoiding the reliance on ineffective antibiotics and underscores the need for alternative treatment options for periodontal diseases [22].

Conversely, the sensitivity of T. denticola to tetracycline and other antibiotics such as imipenem, cefoperazone, chloramphenicol, clindamycin, and moxifloxacin presents a spectrum of potential therapeutic avenues. The varied classes and mechanisms of action of these antibiotics offer flexibility in designing treatment regimens tailored to the susceptibility profile of T. denticola. This diversity in antibiotic sensitivity is particularly significant in the context of developing individualized treatment plans that consider patient-specific factors and the complex microbial environment associated with periodontal disease.

The implications of this research extend beyond the specific susceptibility profile of T. denticola. The study contributes to the broader field of antimicrobial stewardship by emphasizing the importance of tailored treatment strategies based on detailed microbial susceptibility information. This approach aligns with the global efforts to combat antibiotic resistance by promoting judicious and informed use of antimicrobial agents.

Limitations

Some limitations of the study include the relatively small sample size, as well as the single-center design, which may limit the generalizability of the findings. Additionally, the in vitro nature of the antimicrobial susceptibility testing may not fully reflect the complex microbial interactions present in vivo. The selection of the antimicrobial panel could also be expanded to include other agents or combination therapies. Furthermore, while the study focused on Treponema denticola, consideration of interactions with other periodontal pathogens and clinical correlation with treatment outcomes would strengthen the findings. Long-term effects of antimicrobial therapy and potential publication bias are also important factors to consider. Addressing these limitations in future research would provide a more comprehensive understanding of antimicrobial susceptibility in periodontal disease.

## Conclusions

In conclusion, the comprehensive examination of Treponema denticola's antimicrobial susceptibility and resistance profiles sheds light on its role in the complex landscape of periodontal disease. The findings not only enhance our understanding of the interactions between T. denticola and antimicrobial agents but also provide a foundation for refining treatment strategies in the pursuit of more effective and personalized approaches to periodontal care. This research represents a significant step forward in the ongoing endeavor to decipher the intricate microbial dynamics influencing periodontal health and disease.
